# University of Buffalo

**Published:** 1866-02

**Authors:** 


### University of Buffalo.

The commencement exercises of the Medical Department of the University of Buffalo were held at St. James Hall, February 20th. The degree of *Doctor in Medicine* was conferred upon the following gentlemen:

Theophilus St. Vincent Hutchinson, Meadford, Grey county, C.W.
Job Densmore Potter, De Ruyter, Madison county, N. Y.
William McCoy Boice, Meclenburg, Schuyler county, N. Y.
Edwin Washington Marsh, Corfu, Genesee county, N. Y.
Bernhard Kaffenberger, Buffalo, Erie county, N. Y.
James Kennedy, jr., Farmer, Seneca county, N. Y.
John Robinson Alexander, Lancaster, Erie county, N. Y.
Hiram Gettman Dubois, East Florence, Oneida county, N. Y.
George St. John, Short Tract, Alleghany county, N. Y.
George Merrit Beard, Horse Heads, Chemung county, N. Y.
Robert Alexander Gunn, Caledonia, Haldemand county, C.W.
Worp Van Peyma, Lancaster, Erie county, N. Y.
George Washington Nesbitt, Buffalo, Erie county, N. Y.
Eugene N. B. Smith, Buffalo, Erie county, N. Y.
James Henry Johnson, Wilson, Niagara county, N. Y.
Simeon Villermer Pool, Springville, Erie county, N. Y.
Joseph Miller, Varysburg, Wyoming county, N. Y.
Mortimer Robinson Craig, Geneseo, Livingston county, N. Y.
R. Gilbert Platt, Canton, Bradford county, Pa.

Robert J. Menzie, Bergen, Genesee county, N. Y.
Henry Williams Caldwell, Florence, Oneida county, N. Y.
Henry Downer Ingraham, Java, Wyoming county, N. Y.
Conrad Diehl, jr., Buffalo, Erie county, N. Y.
Ernest Lorenzo Shurly, Buffalo, Erie county, N. Y.
Frederick Eugene Hutchinson, Utica, Oneida county, N. Y.
William Cornelius Phelps, Buffalo, Erie county, N. Y.
Orson Clark, Java, Wyoming county, N. Y.
Hiram Forbes Baker, Bellevue, Huron county, Ohio.
Gustavus Engleberth Mackay, Buffalo, Erie county, N. Y.
Marlin Burdett Shaw, Ripley, Chautauqua county, N. Y.
Charles White Morse, Eden, Erie county, N. Y.
Frank Wayland Abbott, Buffalo, Erie county, N. Y.
Eugene Van Buren Cuykendall, Owasco, Cayuga county, N. Y.
Archibald McNeil Bevier, Owasco, Cayuga county, N. Y.
Lewis Blanchard, Centreville, Alleghany county, N. Y.
Edmund Thomas Hewson, Caledonia, Haldemand county, C.W.
Andrew Kamerling, Buffalo, Erie county, N. Y.
John Rolph McCarthy, Black Creek, Welland county, C.W.
Ahira Raymond White, Tonawanda, Erie county, N. Y.
George Dixon Mayne, Maynesville, Pa.

The Honorary Degree of Doctor in Medicine was conferred upon George Swinburn, Rochester, N. Y.

The following gentlemen were elected Curators of the Medical Department of the University:

Charles K. Irwin, M. D., of Dunkirk, N. Y.; George C. Bennett, M. D., of Erie, Pa.; Morris W. Townsend, M. D., of Bergen, N. Y.

